# Three-Dimensional Volumetric Investigation of Onodi Cells: A Multi-Slice Computed Tomography Study

**DOI:** 10.1055/s-0043-1773762

**Published:** 2023-09-26

**Authors:** Flavia Limberg Dieguez, Catharina Simioni De Rosa, Paulo Henrique Braz-Silva, Sergio Lucio Pereira de Castro Lopes, Andre Luiz Ferreira Costa

**Affiliations:** 1 Postgraduate Program in Dentistry, Universidade Cruzeiro do Sul (UNICSUL), São Paulo, SP, Brazil; 2 Division of General Pathology, Department of Stomatology, Faculdade de Odontologia, Universidade de São Paulo (USP), São Paulo, SP, Brazil; 3 Virology Department, Laboratory of Virology, Instituto de Medicina Tropical de São Paulo, Faculdade de Medicina, Universidade deSão Paulo (USP), São Paulo, SP, Brazil; 4 Department of Diagnosis and Surgery, Instituto de Ciência e Tecnologia, Universidade Estadual Paulista (Unifesp), São José dos Campos, SP, Brazil

**Keywords:** paranasal sinuses, diagnostic imaging, three-dimensional imaging, anatomic variation, pneumatization

## Abstract

**Introduction** Onodi cells (OCs) are posterior ethmoid cells that are located above the sphenoid sinus, close to or even surrounding the carotid artery and optic nerve.

**Objective** To investigate and evaluate the volumetric variation of OCs through multi-slice computed tomography (MSCT) scans.

**Methods** We performed a retrospective review of MSCT scans of 79 subjects, 40 male and 39 female patients, Whose age ranged from 18 to 83 (mean: 39.6) years. The volumes of the OCs on the right and left sides were measured using the ITK-SNAP software (open-source) with semiautomatic segmentation. The possible relationships involving age, gender, contact with the optic nerve, extension of the pneumatization of the posterior ethmoid cells into the clinoid processes, mucous thickening in the anterior and posterior ethmoid cells, and obliteration of the sphenoethmoidal complex were analyzed with the Pearson correlation and Chi-squared tests according to the type of data compared and logistic regression models ( *p* < 0.05).

**Results** We observed that an increase of one unit in the volume of OCs also increases the chance of extension of pneumatization into the clinoid processes by 0.15% ( *p* = 0.001). No significant correlations were identified regarding age, gender, and volume of the OCs.

**Conclusion** The volume of the OCs has effects on the extension of pneumatization into the clinoid processes.

## Introduction

The sinus region presents the greatest anatomical variation in the human body, which can cause the sinus ostium or meatus to be narrowed or obliterated. 1 Onodi cells (OCs), or sphenoethmoidal cells, are some of the cellular variations located more superolaterally to the sphenoid sinus, and they are of great practical importance in surgeries due to their close relationship with the optic canal, sphenoid sinus, pituitary fossa, and carotid arteries. 2 Therefore, identifying them is crucial to maximize exposure and reduce the risk of injury to the surrounding structures. 3

In some studies, 3 4 5 the prevalence of OCs varies with different identification methods, such as endoscopy and tomography. This divergence is probably due to the acquisition angle used in an axial computed tomography (CT) scan or to the complexity of image interpretation.

Pneumatization and expansion of the sphenoethmoidal cells are directly linked to the exposure of neurovascular structures (such as the internal carotid artery and the optic canal) during surgical procedures. 6 For instance, the optic nerve may emerge prominently on the lateral wall of these OCs and surround them. 7

Furthermore, the degree of pneumatization impairs mucus drainage and may cause sinonasal mucosal disease. 1

Much has been studied about OCs, but, to the best of our knowledge, there is no study on the impact of their volume on neurovascular structures and mucosal diseases.

The aim of the present study was to investigate the correlation of OC volume with extension of OC pneumatization into the clinoid processes, mucous thickening in anterior and posterior ethmoid cells, and obliteration of the sphenoethmoidal complex.

## Materials and Methods

### Study Sample Selection

We retrospectively selected MSCT scans from an image database of subjects cared for at the Dentomaxillofacial Radiology Division of the School of Dentistry of our institution, who were referred for MSCT scans from March 2020 to March 2021 due to clinical symptoms referable to the sinonasal region. The study was approved by the institutional Ethics in Research Committee, and it was conducted in full accordance with the World Medical Association's 1964 Declaration of Helsinki and later versions.

### Image Acquisition

Images were acquired by using a 4-channel multi-detector CT system (Alexion 4, Canon, Ohta-ku, Tokyo, Japan) in the axial plane, with the patient in supine position and with head in neutral position, without the use of contrast. Volumetric acquisition was performed without angulation and with contiguous 1-mm thick slices and 1-mm intervals (parameters: 100 kV, 100 mA, 1 s/rotation, matrix of 512 × 512 pixels, gap of 0.8 mm, voxel of 0.37 mm × 0.37 mm, and field of view [FOV] of 180 mm × 180 mm) in a bone window (4,000 Hounsfield units [HU]), extending from the nasal process of the maxilla to the apex of the frontal sinus parallel to the hard palate.

A total of five hundred subjects with MSCT scans were investigated, and the image database was searched under anonymous conditions. All the MSCT scans were selected by two senior dentomaxillofacial radiologists according to the following criteria:

*Inclusion criteria* : subjects older than 18 years of age with images showing the middle and upper regions of the face.

*Exclusion criteria* : subjects who had previously undergone nasal or paranasal sinus surgery; who had tumors, fractures, and inflammatory processes altering the continuity of the walls of the posterior ethmoid sinuses; and whose images showed artifacts or distortions in the region of interest due to the patient's movements during image acquisition.

### Image Processing and Volume Analysis

All images were obtained in Digital Imaging and Communications in Medicine (DICOM) format and then exported to the ITK/SNAP software (open source), version 3.8.0, before being selected. 8

**Fig. 1:**
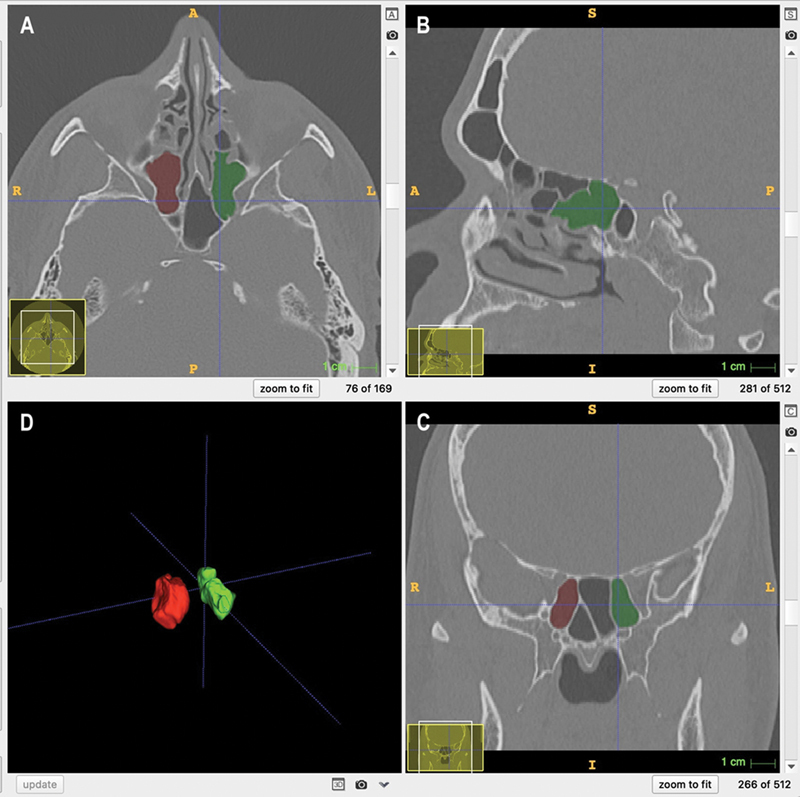
Image showing multiple planar views ( **A** : axial; **B** : sagittal; and **C** : coroanl) with segmentation and 3D surface model of Onodi cells displaying volume rendering ( **D** ).

A radiologist with experience in MSCT imaging identified and segmented all the OCs independently, and a semiautomatic method was used by making the active contour evolving toward the target object to define the region of interest (ROI) and threshold based on air bubbles inside the OCs, which were identified as the lateral extension of posterior ethmoid cells. 9 The left and right maxillary sinuses were marked with different colors to calculate the volumes separately. The ITK/SNAP software enabled the visualization of the three orthogonal planes and the three-dimensional reconstructed object ( Fig. 1 ).

### Statistical Analysis

Exploratory data analysis was performed through summary measures (such as frequency, percentage, mean, standard deviation, median, and minimum and maximum values) and graphs developed with the R (R Foundation for Statistical Computing, Vienna, Austria), version software 4.1.1. 10 The intraclass correlation coefficient (ICC) was used to evaluate the repeatability of volume measurements, whereas the Pearson correlation coefficient, to assess the correlation between age and volume. The Student *t* -test was used to compare age between bilateral and unilateral groups, and the Chi-squared test, to compare the bilaterality among categorical variables. The total volume was used to assess the influence of OC volume on age, gender, contact with the optic nerve, extension of the pneumatization of the posterior ethmoid cells into the clinoid processes, mucous thickening in anterior and posterior ethmoid cells, and obliteration of the sphenoethmoidal complex, that is, the sum of the volume is for bilateral subjects, and the volume of the side with OC is for unilateral subjects. Logistic regression models were used to assess the chance of occurrence of outcomes depending on the volume, adjusted for bilaterality. The significance level adopted was of 5%.

## Results

**Fig. 2:**
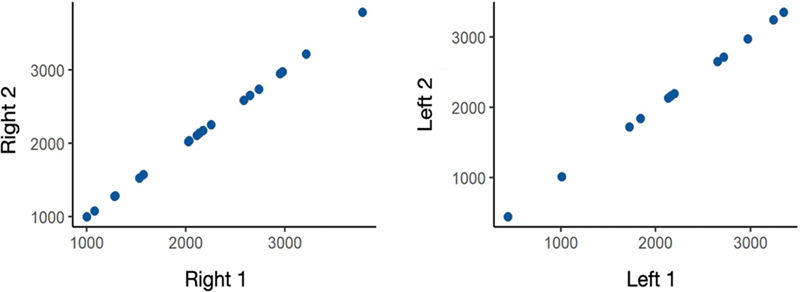
Scatter plots between the two measurements at the two moments studied.

According to the eligibility criteria, 79 exams were included, 65 of which were from the right side, 46, from the left side, and 39, bilateral. These 79 exams are shown in Table 1 , and Fig. 2 shows the evaluation of the repeatability of measurements at 2 different moments. There was an excellent agreement between the two moments, with the ICC very close to 1. The age of the patients ranged from 18 to 83 years, with a mean age of 39.6 years and standard deviation of 16.1 years. The correlation observed between age and volume was low ( *ρ* = 0.102).

**Table 1:** Measurements of position and volume dispersion at the two moments on each side and intraclass correlation coefficient (ICC)

Side	Mean	Standard deviation	Minimum	Median	Maximum	ICC
Right 1	2,179	768	1,001	2,139	3,784	0.999
Right 2	2,203	871	442	2,179	3,349	
Left 1	2,179	768	1,003	2,141	3,785	0.999
Left 2	2,203	871	442	2,179	3,351	

**Fig. 3:**
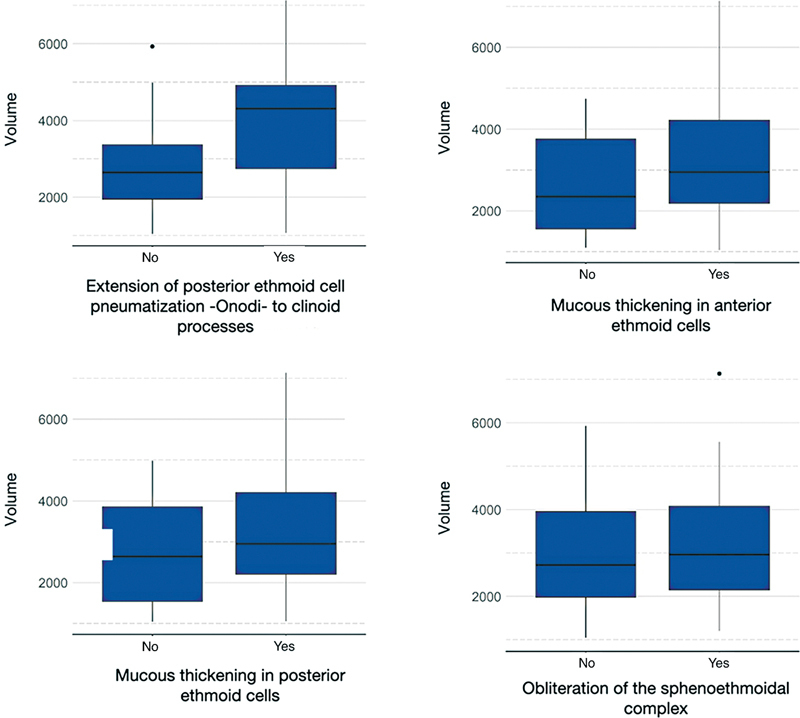
Boxplot identifying the volume of the variables analyzed.

Table 2 presents the descriptive measurements of the total volume for each gender, contact with optic nerve, and other outcomes. The results were not statistically significant ( *p* < 0.05) for any of the variables. Fig. 3 presents the boxplot of the total volume *per* variable.

**Table 2:** Measurements of position and volume dispersion of Onodi cells

Variables	Rank	Mean	Standard deviation	Minimum	Median	Maximum
Gender	Female	3,062	1,274	1,078	2,948	5,559
	Male	2,990	1,425	1,039	2,744	7,133
Contact with the optic nerve	No	3,307	1,415	1,079	2,744	5,498
	Yes	2,941	1,324	1,039	2,942	7,133
Extension of the pneumatization of Onodi cells into the clinoid processes	No	2,714	1,146	1,039	2,648	5,932
	Yes	3,993	1,485	1,076	4,308	7,133
Mucous thickening in the anterior ethmoid cells	No	2,630	1,220	1,101	2,346	4,740
	Yes	3,120	1,366	1,039	2,951	7,133
Mucous thickening in the posterior ethmoid cells	No	2,729	1,346	1,039	2,637	4,984
	Yes	3,135	1,340	1,059	2,951	7,133
Obliteration of the sphenoethmoidal complex	No	2,937	1,327	1,039	2,724	5,932
	Yes	3,192	1,389	1,206	2,962	7,133

Table 3 shows the comparison between the bilateral and unilateral groups regarding age, gender, contact with the optic nerve, and the other variables. Once more, no statistically significant differences were observed between the groups for any of the variables evaluated.

**Table 3:** Measurements of position and volume dispersion of Onodi cells

Variables	Bilateral		*p* -value
	No ( *N* = 39)	Yes ( *N* = 39)	
Age in years	37.5 (14.4)	41.7 (17.5)	0.250
Gender			
* Female*	17 (43.6%)	21 (53.8%)	0.497
* Male*	22 (56.4%)	18 (46.2%)	
Contact with the optic nerve			
* No*	10 (25.6%)	8 (20.5%)	0.788
* Yes*	29 (74.4%)	31 (79.5%)	
Extension of the pneumatization of Onodi cells into the clinoid processes			
* No*	32 (82.1%)	27 (69.2%)	0.291
* Yes*	7 (17.9%)	12 (30.8%)	
Mucous thickening in the anterior ethmoid cells			
* No*	9 (23.1%)	6 (15.4%)	0.566
* Yes*	30 (76.9%)	33 (84.6%)	
Mucous thickening in the posterior ethmoid cells			
* No*	12 (30.8%)	9 (23.1%)	0.610
* Yes*	27 (69.2%)	30 (76.9%)	
Obliteration of the sphenoethmoidal complex			
* No*	26 (66.7%)	25 (64.1%)	1.000
* Yes*	13 (33.3%)	14 (35.9%)	

Table 4 presents the results of the logistic regression models for the influence of OC volume on the four variables studied: extension of the pneumatization of the posterior ethmoid cells (OCs) to the clinoid processes; mucous thickening the in anterior ethmoid cells; mucous thickening in the posterior ethmoid cells; and obliteration of the sphenoethmoidal complex. The volume used is the sum of the two sides, and the models were adjusted for bilaterality. We observed that an increase of 1 mm ^3^ in volume, adjusted for bilaterality, increases the chance of extension of pneumatization by 0.15% ( *p* = 0.001), whereas an increase of 1 cm ^3^ in total volume increases the chance of extension of pneumatization by 15.7%.

**Table 4:** Odds ratios (ORs) for the influence of volume on outcomes calculated by logistic regression

Variables	OR*	95% confidence interval (OR)	*p* -value
Extension of the pneumatization of Onodi cells into the clinoid processes	**1.0015**	**1.0007–1.0025**	**0.001**
Mucous thickening in the anterior ethmoid cells	1.0003	0.9997–1.0011	0.328
Mucous thickening in the posterior ethmoid cells	1.0003	0.9997–1.0010	0.347
Obliteration of the sphenoethmoidal complex	1.0002	0.9997–1.0008	0.359

Notes: Adjusted for bilaterality. Values in bold are significant ( *p* < 0.05).

## Discussion

As this region presents the greatest anatomical variation in the human body, 1 several studies have already highlighted the importance of precisely identifying ethmoid cells, 2 4 5 particularly the OCs, also known as sphenoethmoidal air cells. 6 7

In a recent article, 8 the authors reported that OCs can have a high variability of prevalence, ranging from 1.6% to 55.8%. We can assume that the high prevalence may be associated with the fact that several different devices can be used to acquire the images, such as CT scanners, 11 cone beam CT scanners, 3 and magnetic resonance imaging scanners. 9 These devices provide different types of visualization in terms of precision. Based on the literature, 1 2 3 6 10 12 we chose to use images from MSCT scans because they are the most comfortable exam for the patient and provide good-quality images of bony structures in the paranasal sinus region.

Results from earlier studies 9 11 12 13 14 indicated that there is an equal distribution between genders regarding the anatomic variation of OCs. The present study is in agreement with these results, as we found no statistically significant association between cell volume and gender for the unilateral and bilateral groups.

Previous studies 9 10 11 12 have reported an age range from 8 to 85 years, with a mean age between the third and fourth decades of life. The present study corroborates this finding. Despite the age variability, we found no statistically significant association between cell volume and age for the unilateral and bilateral groups.

It should be emphasized that the formation of each paranasal sinus is different in terms of shape and size, meaning that each of them has its own characteristics in children and adults. For instance, the ethmoid sinus is present since the child's birth. From the age of 8 years onwards, pneumatization of the ethmoid cells progresses posteriorly until the lateral and medial walls are no longer at the same level and become parallel. 11

The first variable investigated was the contact with the optic nerve. The identification of OCs is very important clinically because of their proximity to the optic nerve canal. 2 6 8 12 Most authors would agree that there is a positive correlation between OCs and the optic nerve. 6 8 Chmielik and Chmielik 14 demonstrated that the wall of the optic nerve canal was in contact with at least one posterior ethmoid cell (55.6%). Mazzurco et al. 9 and Lee and Au 15 reported two patients who had optic neuropathy and temporary visual loss due to the presence of OC inflammation, thus highlighting the importance of knowing the proximity of OCs to neighboring anatomic structures. However, the present study contrasts with their findings, as we did not observe statistically significant associations between OC volume and contact with the optic nerve for the unilateral and bilateral groups.

The anterior clinoid process is a delicate area contiguous to the sphenoid bone and optic canal. Many of the literature findings confirm that there is a significant correlation between the posterior clinoid process and OCs on both sides. 11 12 Although the present study demonstrated such a correlation unilaterally, we found no significant association between OC volume and the posterior clinoid process. Nevertheless, when we used logistic regression and adjusted the sample for bilaterality, we found a statistically significant association ( *p* = 0.001), which is consistent with the literature.

Pneumatization of the clinoid process is considered a critical concern in surgery of the base of the skull. The air cells in a pneumatized clinoid process enable communication with the paranasal sinuses. During a clinoidectomy, there may be an opening of the paranasal sinuses, leading to the risk of rhinorrhea and high probability of sepsis. 16

Mucous thickening is characterized by an inflammatory reaction with hyperplasia of the mucous lining of the maxillary sinus, which can be observed on a CT scan as a hypodense structure, sometimes oval. When these structures are observed in the paranasal regions, it may be suggestive of sinusitis or rhinosinusitis. 17 However, the present study was comparable to a previous work, 18 which suggests that there was no association of OCs with mucous thickening or with sphenoid sinusitis and rhinosinusitis. 12 Moreover, subsequent studies showed the presence of OC mucocele 15 17 19 potentially associated with fungal ball. 20

Finally, obliteration of the sphenoethmoidal complex was the last variable investigated. The results found by Doubi et al. 13 show the importance of carefully studying this area, as the presence of OCs can displace the sphenoid sinus ostium inferiorly. However, we observed no statistically significant association between OC volume and obliteration of the sphenoethmoidal complex in the unilateral and bilateral groups.

The main limitation of the present study is that it was conducted retrospectively. Another limitation is that the semiautomatic segmentation was only performed by one examiner, although our sample was measured at two different moments and presented a very strong ICC, which decreased the bias.

Understanding the paranasal region during the clinical practice is vital for surgical procedures and early diagnoses. To our knowledge, the present was the first study on the correlation of OC volume with the extension of pneumatization of posterior OCs into the clinoid processes, mucous thickening in anterior and posterior ethmoid cells, and obliteration of the sphenoethmoidal complex.

## Conclusion

The present study offers supplementary volume data for the anatomical characterization of OCs, showing that the increase in cell volume has effects on the extension of their pneumatization into the clinoid processes.
